# High-Flow Nasal Cannula in COVID-19 Patients With Moderate to Severe Respiratory Distress: A Retrospective Analysis

**DOI:** 10.7759/cureus.52518

**Published:** 2024-01-18

**Authors:** Lubna Saffarini, Nour Sabobeh, Chafika Lasfer, Sara Kazim

**Affiliations:** 1 Emergency Department, Rashid Hospital, Dubai, ARE; 2 Emergency Medicine Department, Fakeeh University Hospital, Dubai, ARE

**Keywords:** high-flow nasal cannula (hfnc), covid-19, oxygen support, rox index, respiratory distress

## Abstract

Background: A high-flow nasal cannula (HFNC) is a device for non-invasive ventilation (NIV). It was utilized during the COVID-19 pandemic in patients with moderate to severe respiratory distress due to its benefit profile in delaying intubation, ease of use, and comfort of patients in comparison to NIV.

Objectives: Our main objective is to calculate the intubation rate of patients with suspected or lab-confirmed COVID-19 in moderate to severe respiratory distress who failed graded oxygen therapy (GOT). Before incorporating HFNC as a treatment option, the intubation rate was 100% after GOT failure. We calculated the rate of intubation at two, six, and 12 hours of starting HFNC, where each patient is in their own control with an assumed intubation rate of 100%. Other objectives include measuring the rate of improvement of the ROX index, respiratory rate (RR), and oxygen saturation (SPO2) levels at two, six, and 12 hours.

Methods: We retrospectively screened patients with suspected or lab-confirmed COVID-19 infection in moderate to severe respiratory distress at Rashid Hospital Trauma Center, Emergency Department in Dubai, United Arab Emirates, from April 10, 2020, until December 31, 2020. The list of patients was pooled from the SALAMA electronic system.

Results: A total of 121 patients were included in the analysis. Assuming an intubation rate of 100% at 0 hours (end of GOT), after starting HFNC, the intubation rate (primary outcome) at two hours was 7.43% (9/121), at six hours was 7.14% (8/112), and at 12 hours was 5.77% (6/104). The total intubation rate at 12 hours was 19% (23/121). The use of HFNC was also shown to improve the ROX index, RR, and SPO2 at two, six, and 12 hours.

Conclusion: In patients with suspected or lab-confirmed COVID-19 in moderate to severe respiratory distress who failed GOT and were started on HFNC, it was noted that the intubation rate decreased from an assumed rate of 100% to 19% at 12 hours from starting the treatment. There was also a statistically significant improvement in the ROX index, SPO2, and RR at two, six, and 12 hours from the initiation at 0 hours.

## Introduction

A high-flow nasal cannula (HFNC) is a device used for non-invasive ventilation (NIV) of patients in moderate to severe respiratory distress. The advantages of this device pertain to patient outcome and comfort by delivering small amounts of positive pressure as well as larger flow rates of oxygen per minute. It can decrease dead space and improve oxygenation [1]. Furthermore, in contrast to NIV machines, continuous positive airway pressure (CPAP), and bilevel positive airway pressure (BiPAP), HFNC delivers heated and humidified air and allows patients to suction, speak, and eat while wearing the device, making it more comfortable for patients and allowing them to tolerate it for longer periods of time. Its positive effects were initially documented in infants by Shoemaker et al. in a retrospective study where they compared the effects of nasal CPAP to HFNC [2]. They reported that more infants were intubated for failing early CPAP compared to early HFNC (40% to 18%) [2].

The advantages of HFNC oxygenation have already been established in the literature in select patients. Frat et al. demonstrated a reduced requirement for intubation in patients with a PaO2/FiO2 ratio <200 and was associated with a reduction in mortality when compared with an NIV or regular oxygen face mask [3]. They prospectively assessed patients with acute hypoxemic respiratory failure and compared conventional oxygen therapy with HFNC and NIV [3]. With an intention to treat analysis, it was suggested that HFNC had the lowest percentage of intubation rates (38%) in comparison to conventional oxygen therapy (47%) and NIV (50%), although it was statistically insignificant [3]. Despite that, there was a significant difference in favor of HFNC in 90-day mortality in comparison to the other groups, with a hazard ratio of more than 2 [3].

Another study by Stéphan et al. did a non-inferiority randomized clinical trial of a total of 830 patients comparing HFNC to BiPAP [4]. With an intention to treat analysis, the primary outcome of the study was met, and it was noted that HFNC is non-inferior to BiPAP [4]. Another systematic review and meta-analysis study by Rochwerg et al. stated that HFNC in acute hypoxemic respiratory failure did not affect mortality rates; however, it significantly reduced the risk of requiring intubation or escalation of oxygen therapy in comparison to conventional oxygen therapy [5]. In 2019, the ROX index was developed and proposed by Roca et al. as a bedside tool to predict the success or failure of HFNC therapy by utilizing objective measurements of oxygen saturation (SPO2), respiratory rate (RR), and FiO2 at two, six, and 12 hours of therapy [6]. They reported that a ROX index of ≥4.88 measured after HFNC therapy at two, six, and 12 hours was associated with a lower risk of HFNC failure [6].

Recently published guidelines regarding the treatment of COVID-19 patients have supported the use of HFNC oxygen therapy. The surviving sepsis campaign has advocated for the use of HFNC for COVID-19-positive patients in acute hypoxemic respiratory failure over conventional oxygen therapy [7]. The American College of Physicians gave a recommendation level 1a where it suggested that “clinicians use HFNC rather than NIV in hospitalized adults for the management of acute hypoxemic respiratory failure” [8]. The European Society of Intensive Care Medicine also made a “strong recommendation for HFNC in hypoxemic respiratory failure compared to conventional oxygen therapy” [9]. The European Respiratory Society also released in their clinical guidelines a recommendation for the use of HFNC in acute respiratory failure, suggesting that it has an advantage over conventional oxygen therapy and NIV [10].

If used correctly, HFNC can be utilized as a tool to improve patient outcomes, decrease the need for intubation, and reduce mortality in COVID-19-positive patients. We conducted a retrospective analysis involving COVID-19 patients with moderate to severe respiratory distress who failed graded oxygen therapy (GOT) and were started on HFNC to study its impact on reducing intubation rates in our population and improving other variables such as the ROX index, RR, and SPO2 levels.

## Materials and methods

Ethical approval

We conducted the study in Rashid Hospital Trauma Center’s Emergency Department (RHTC ED), Dubai Health Authority (Dubai Health now), United Arab Emirates. The study protocol was approved by the Dubai Scientific Research Ethics Committee (approval number: DSREC-07/2021_04). An informed consent was not obtained as the study is a retrospective analysis, and it was waived by the ethical committee. A patient confidentiality form was also signed and submitted. Data was collected through the SALAMA Electronic System, and it was plotted in an Excel sheet (Microsoft Corporation, WA, USA).

Data collection

The data was collected through the SALAMA Electronic System of patients with the following keywords: coronavirus, COVID-19, covid pneumonia, and zone 4 (a negative pressure treatment area in the emergency department designated for COVID-19 patients who require oxygen support) from April 10, 2020, to December 31, 2020. The files were checked, and data collected from the ED physician notes (primary assessment of patient, reason for starting GOT, HFNC, and/or intubation, reassessment of patients), nursing flow sheets (vital signs on arrival and then hourly, start and end time of GOT, HFNC, and/or intubation), respiratory therapists' notes (FiO2, SPO2, and RR levels) and lab results (arterial blood gas (ABG) results).

Inclusion and exclusion

Patients included in this study were adults aged 18 and above presenting to the RHTC ED with suspected or lab-confirmed COVID-19 in moderate to severe respiratory distress who failed GOT. GOT success was defined as using a nasal cannula (NC) at 5-6 L/min and a non-rebreather mask at 15 L/min to maintain a target SPO2 of >90%. GOT failure is defined as SPO2 <90%, RR >30/min, unable to speak full sentences, heart rate >130/min, or a PaO2:FiO2 <300. Patients excluded from the study are patients who were intubated without an HFNC trial, patients with no oxygen requirements, chronic obstructive pulmonary disease and asthmatic patients, cardiogenic pulmonary edema patients, patients who were agitated, uncooperative, or found to have a low Glasgow coma scale, and children <18 years of age. Lab-confirmed COVID-19 is defined as a positive result obtained through a COVID-19 RNA-PCR swab using the gene expert module and collected via the nasopharyngeal or oropharyngeal route. Suspected COVID-19 patients were defined as patients presenting with upper or lower respiratory symptoms with or without fever and any of the following: history of international travel 14 days prior to symptom onset; close contact with a confirmed case of COVID-19 within 14 days; residing in a community setting where COVID-19 cases were detected, including healthcare facilities; or cases of fever and cough without a history of travel or known possible exposure.

Characteristics of patients

The characteristics of patients at baseline in our study were identified at the time of presentation to the ED prior to any intervention (Table 1). The mean age was 52 years old, with a standard deviation of 11.536. Our population was predominantly male (76.9%), and 38.8% were of Indian descent. The median baseline SPO2 in room air was 85% with a minimum of 30% and a maximum of 99%, while the median baseline RR was 26 bpm with a minimum of 17 bpm and a maximum of 42 bpm. Fifty-four percent were diabetic, 58% were hypertensive, and 8% were documented to be smokers. Characteristics at 0 hours were those at the end of GOT (Table 2). Tables 3-5 indicate the change over time in the characteristics of patients after the initiation of HFNC at two, six, and 12 hours, respectively.

**Table 1:** Characteristics of patients at baseline n: number of patients, no.: number, M: male, F: female, CARP: COVID awake repositioning and proning, SPO2: oxygen saturation, RR: respiratory rate, SBP: systolic blood pressure, DBP: diastolic blood pressure, mmHg: millimeters of mercury, Temp: temperature, *: mean and standard deviation, **: median and range

Characteristics of patients at baseline	n=121	On initial assessment and before GOT (room air)
Age (years)	121	52.5 (11.536)*
Sex - no. (%)	121	M = 93 (76.9), F = 28 (23)
Nationality - no. (%)	121	India = 47 (38.8), Pakistan = 20 (16.5), Philippines = 16 (13.2), others = 38 (31.4)
Diabetes mellitus - no. (%)	121	54 (44.6)
Hypertension - no. (%)	121	58 (47.9)
Smoking - no. (%)	121	8 (6.6)
CARP - no. (%)	121	110 (90.9)
SPO2 (%)	121	85 (30-99)**
RR (breaths per minute)	121	26 (17-42)**
SBP (mmHg)	121	131 (20.454)*
DBP (mmHg)	121	79 (42-142)**
Pulse (beats per minute)	121	106.5 (19.533)*
Temp (degrees Celsius)	121	38 (36.4-40.5)**

**Table 2:** Characteristics of patients at 0 hours n: number of patients, no.: number, GOT: graded oxygen therapy, SPO2: oxygen saturation, bpm: breaths per minute, PaO2: partial concentration of oxygen, mmHg: millimeters of mercury, FiO2: fraction of inspired oxygen, PaO2:FiO2: partial concentration of oxygen to fraction of inspired oxygen ratio, ROXI: ROX index, CARP: COVID awake repositioning and proning, *: mean and standard deviation, **: median and range

Characteristics of patients at 0 hours	n=121	End of GOT
SPO2 (%)	121	88 (64-99)**
RR (bpm)	121	30 (18-60)**
Pulse (beats per minute)	121	96.88 (18.352)*
PaO2 (mmHg)	26	58.35 (10.508)*
FiO2 (no.)	121	1 (0.5-1)**
PaO2:FiO2 (no.)	26	0.58 (0.105)*
ROXI (no.)	121	2.933 (1.550-5.11)**
CARP (%)	110	90.9

**Table 3:** Characteristics of patients at two hours n: number of patients, no.: number, SPO2: oxygen saturation, bpm: breaths per minute, PaO2: partial concentration of oxygen, mmHg: millimeters of mercury, FiO2: fraction of inspired oxygen, PaO2:FiO2: partial concentration of oxygen to fraction of inspired oxygen ratio, ROXI: ROX index, CARP: COVID awake repositioning and proning, *: mean and standard deviation, **: median and range

Characteristics of patients at 2 hours	n=121	2 hours from HFNC initiation
SPO2 (%)	120	93 (58-100)**
RR (bpm)	120	28 (16-49)**
Pulse (beats per minute)	120	89.5 (59-152)**
PaO2 (mmHg)	15	53.10 (39-142)**
FiO2 (no.)	119	0.9 (0.3-1)**
PaO2:FiO2	15	61.66 (44.884)*
ROXI (no.)	119	3.76 (1.708-15.84)**
CARP (%)	119	98.3
Intubation (%)	9	7.43

**Table 4:** Characteristics of patients at six hours n: number of patients, no.: number, SPO2: oxygen saturation, bpm: breaths per minute, PaO2: partial concentration of oxygen, mmHg: millimeters of mercury, FiO2: fraction of inspired oxygen, PaO2:FiO2: partial concentration of oxygen to fraction of inspired oxygen ratio, ROXI: ROX index, CARP: COVID awake repositioning and proning, *: mean and standard deviation, **: median and range

Characteristics of patients at 6 hours	n=112	6 hours from HFNC initiation
SPO2 (%)	106	93 (77-100)**
RR (bpm)	106	26 (16-45)**
Pulse (beats per minute)	105	87.48 (16.354)*
PaO2 (mmHg)	6	59.516 (13.081)*
FiO2 (no.)	105	0.9 (0.4-1)**
PaO2:FiO2 (no.)	6	47.87 (39.084)*
ROXI (no.)	105	3.92 (2-15)**
CARP (%)	103	91.9
Intubation (%)	8	7.14

**Table 5:** Characteristics of patients at 12 hours n: number of patients, no.: number, SPO2: oxygen saturation, bpm: breaths per minute, PaO2: partial concentration of oxygen, mmHg: millimeters of mercury, FiO2: fraction of inspired oxygen, PaO2:FiO2: partial concentration of oxygen to fraction of inspired oxygen ratio, ROXI: ROX index, CARP: COVID awake repositioning and proning, *: mean and standard deviation, **: median and range

Characteristics of patients at 12 hours	n=104	12 hours from HFNC initiation
SPO2 (%)	97	94 (78-100)**
RR (bpm)	97	26.23 (6.204)*
Pulse (beats per minute)	97	83.55 (13.791)*
PaO2 (mmHg)	6	53.88 (9.752)*
FiO2 (no.)	97	0.9 (0.4-1)**
PaO2:FiO2 (no.)	6	53.99 (33.994)*
ROXI (no.)	97	4.08 (2.21-19.2)**
CARP (%)	100	96.15
Intubation (%)	6	5.77

Statistical analysis

Numerical data were reported as mean +/- standard deviation for bell-shaped data and median with range for skewed data. Categorical variables were presented as counts and percentages. We used the Wilcoxon signed-rank test to compare the numerical variables at different points compared with 0 hours before the initiation of HFNC. A p-value of <0.05 was deemed statistically significant.

## Results

**Figure 1:**
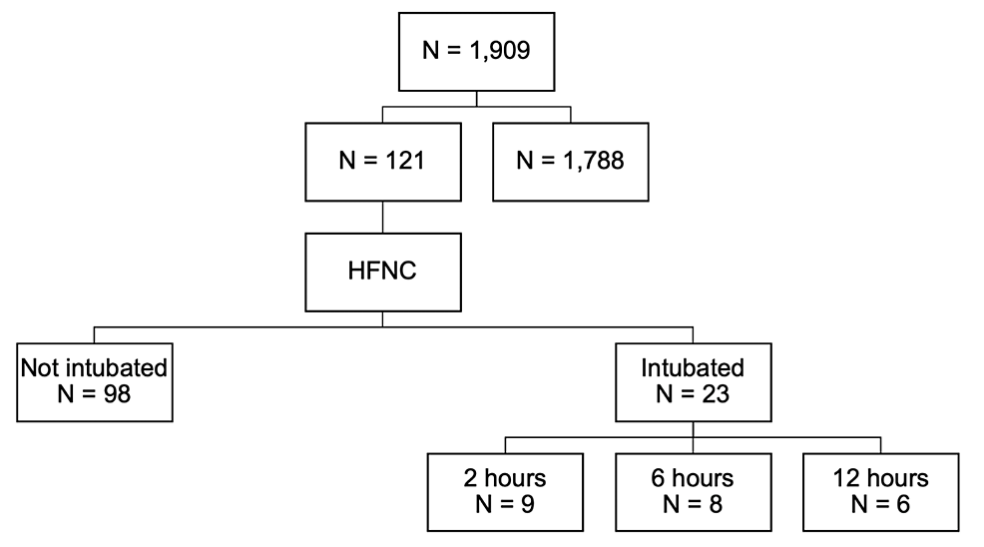
Flowchart of patient screening for eligibility N: number of patients, HFNC: high flow nasal cannula

In total, 1,909 patients were screened for eligibility, of which 121 patients experiencing moderate to severe respiratory distress who were started on GOT experienced failure (Figure 1).

Intubation

**Figure 2:**
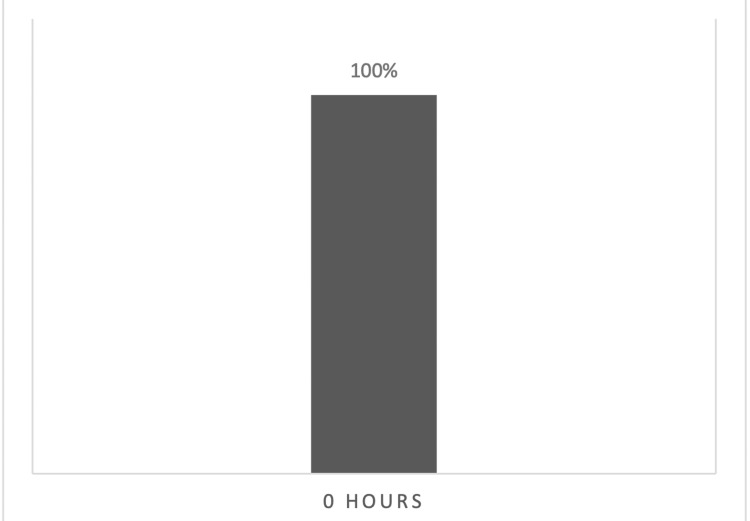
Assumed intubation rate after GOT GOT: graded oxygen therapy

**Figure 3:**
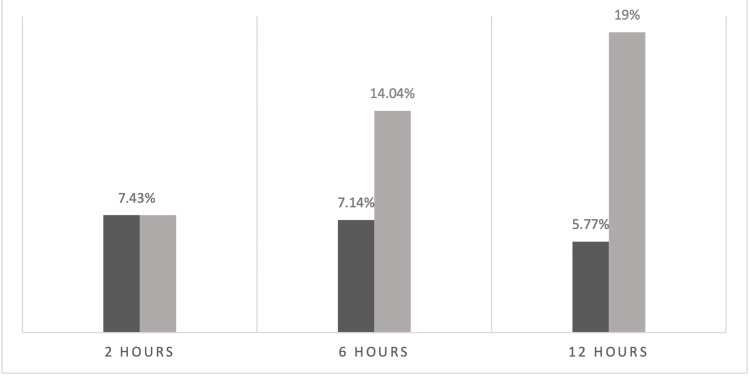
Intubation rates after starting HFNC HFNC: high-flow nasal cannula At two hours: the dark and light grey columns are nine patients intubated out of 121 patients At six hours: the dark grey column is eight patients intubated out of 112, and the light grey column is the cumulative rate of 17 patients intubated out of 121 patients At 12 hours: the dark grey column is six patients intubated out of 112, and the light grey column is the cumulative rate of 23 patients intubated out of 121 patients

With patients acting as their own control and assuming an intubation rate of 100% at 0 hours (Figure 2), all were started on HFNC and monitored at two, six, and 12 hours. Out of 121 patients on HFNC, nine patients (7.43%) were intubated at two hours, eight (7.14%) out of 112 patients were intubated at six hours, and six (5.77%) out of the remaining 104 patients at 12 hours were intubated (Figure 3). A cumulative intubation rate of 14.04% at six hours (17 out of 121 patients) and 19% at 12 hours (23 out of 121 patients) is noted (Figure 3), which indicates a decrease in the intubation rates of COVID-19 patients in moderate to severe respiratory distress after starting HFNC.

ROX index

The ROX index was measured for all 121 patients at 0 hours with a median of 2.933 (range: 1.550-5.111). At two hours, it was measured for 119 patients out of 121, and an improvement was noted to a median of 3.7600 (range: 1.7083-15.840) (p≤0.001 for comparison with 0 hours). At six hours, it was measured for 105 patients out of 112 with an improvement to a median of 3.92 (range: 2-15) (p<0.001 for comparison with 0 hours), and at 12 hours, the median improved further to 4.08 (range: 2-19) (p≤0.001 for comparison with 0 hours), yet it was only measured for 96 out of 104 patients.

SPO2

The SPO2 was measured for all 121 patients at 0 hours with a median of 88 (range: 64-99). At two hours, it was measured for 120 patients out of 121, and an improvement was noted to a median of 93 (range: 58-100). At six hours, it was measured for 106 patients out of 112 with the same median of 93 (range: 77-100). Finally, at 12 hours, it was measured for 97 patients out of 104 with a median of 94 (range: 78-100). The p-value for comparison between the SPO2 levels at two, six, and 12 hours and 0 hours was <0.001.

RR

The RR was measured for all 121 patients at 0 hours with a median of 30 bpm (range: 18-60). At two hours, it was measured for 120 patients out of 121, with a median of 28 (range: 16-49). It was measured for 106 patients out of 112 at six hours, with a median of 26 (range: 16-45). At 12 hours, it was only measured for 97 out of 104 patients, with a mean of 26.23 (standard deviation 6.204). The p-value for comparison between the RR at two, six, and 12 hours with 0 hours was <0.001.

## Discussion

In our single-center retrospective analysis, HFNC reduced the intubation rates to 7.43% at two hours, 11.5% at six hours, and 19% at 12 hours in COVID-19 patients with moderate to severe respiratory distress who failed GOT. When planning the study, we assumed an intubation rate of 100% in patients who failed a trial of GOT based on our clinical practice in RHTC prior to the application of HFNC in the clinical practice guideline for COVID-19 patients.

Demoule et al. noted a decrease in intubation rates by 55% in critically ill COVID-19 patients [11]. A meta-analysis and systematic review by Li et al. indicated that patients with COVID-19 treated with HFNC had a statistically significant lower intubation rate compared with those undergoing conventional oxygen therapy (nasal cannula, simple, or venturi face mask) [12]. Wang et al. also reported a 41% failure rate of HFNC in COVID-19 patients in comparison to a 59% success despite a small sample size [13]. A literature review by Gürün Kaya et al. found that HFNC can reduce the need for intubation in patients with COVID-19 [14]. A randomized, open-label clinical trial conducted in the EDs and ICUs of three hospitals in Colombia indicated that HFNC in patients with severe COVID-19 significantly decreased the need for intubation, mechanical ventilation support, and time to clinical recovery compared with conventional low-flow oxygen therapy [15]. Alshahrani et al. concluded from a prospective cohort study that one-third of hypoxemic COVID-19 ICU patients who received HFNC did not require intubation [16]. Generally, there is a consistent positive effect of HFNC in delaying and reducing intubation rates in COVID-19 patients and patients with respiratory failure, and the former is in congruence with our findings.

Our study also demonstrated a persistent improvement of the ROX index after HFNC initiation at two hours (median 2.933), six hours (median 3.92), and 12 hours (median 4.08). Our findings are consistent with other studies; for example, Hanci et al. showed there was an overall increase in the ROX index of ICU patients with COVID-19 respiratory failure who were subsequently weaned from HFNC with a p-value of 0.002 [17]. Chandel et al. documented that a ROX index of more than 3.67 12 hours after the application of HFNC was an accurate predictor of successful weaning [18]. Another study by Duan et al. evaluated the success of HFNC in relation to ROX index values in COVID-19 patients and showed that the ROX index tends to increase from the first hour of treatment in the success group [19]. A study by Xu et al. stated that a lower ROX index is associated with an increased HFNC failure rate, and it has a high sensitivity in identifying HFNC failure in COVID-19 patients [20]. Alshahrani et al. also found that a low ROX index was associated with HFNC failure, and a ROX index of ≥4.88 at two hours showed lower intubation rates [16]. The same finding was noted at six, 12, and 18 hours [16].

As for the RR and SPO2 levels, our study showed a constant improvement in both after the initiation of HFNC. One study by Hirabayashi et al. showed that HFNC failure patients with COVID-19 had a higher RR [21]. In Wang et al.’s study, the RR was found to decrease significantly after one to two hours of HFNC use in successful cases [13]. The study by Alshahrani et al. found that the RR was lower after 24 hours of HFNC initiation in the group of patients who benefited from it, at a median of 21 breaths per minute [16]. Furthermore, Blez et al. demonstrated that an RR ≥26 breaths per minute after 30 minutes of HFNC is associated with a greater risk of HFNC failure, despite the study having a small sample size of 30 [22]. Another study found that low SpO2 levels at admission were a predictor of HFNC failure [23]. Sztrymf et al.’s study also revealed that HFNC significantly reduced the RR and increased pulse oximetry in ICU patients with acute respiratory failure [24].

Strengths and limitations

Some of the strengths of our retrospective analysis are strict adherence to our inclusion and exclusion criteria, screening a large pool of patients, and a comprehensive literature review. However, the study has a lot of limitations. Our sample size is small despite screening 1,909 patient files; it is a single-center observational study in one ED; the patients were their own control; and unfortunately, not all variables were documented, hence why some parameters are missing, which biases the results. For example, a full set of vitals was not documented for all patients, so some SPO2 and RR levels were missing; the FiO2 was also not regularly documented, which affects the calculation of the ROX index; and not all patients had ABGs done for them, so the PaO2 and PaO2:FiO2 ratio values were missing for some patients. Future research should focus more on the ROX index as a measure to avoid intubation, benefit from HFNC treatment, and assess hospital length of stay and mortality benefit.

## Conclusions

In this retrospective single-center analysis, it was noted that the cumulative intubation decreased from an assumed rate of 100% at the end of GOT to 7.43% at two hours (9/121), 14.04% at six hours (17/121), and 19% at 12 hours (23/121) in moderate to severe COVID-19 patients after HFNC initiation. A statistically significant and persistent improvement in the ROX index was also noted, from a median of 2.933 at 0 hours to 3.76 at two hours, 3.92 at six hours, and 4.08 at 12 hours. There was also a statistically significant improvement in the RR and SPO2 levels at two hours, six hours, and 12 hours in comparison to 0 hours.
